# Effect of low-level light therapy in patients with dry eye: a prospective, randomized, observer-masked trial

**DOI:** 10.1038/s41598-022-07427-6

**Published:** 2022-03-04

**Authors:** Yuli Park, Hoon Kim, Sehwan Kim, Kyong Jin Cho

**Affiliations:** 1 Department of Ophthalmology, Dankook University Hospital, College of Medicine, Dankook University, Cheonan, Republic of Korea; 2 Department of Biomedical Engineering, School of Medicine, Dankook University, Cheonan, Republic of Korea

**Keywords:** Diseases, Medical research

## Abstract

To evaluate the efficacy of low-level light therapy (LLLT) with near-infrared light-emitting diodes (LED-LLLT) for the treatment of dry eye. 40 patients were randomly assigned with a 1:1 allocation ratio to receive LED-LLLT (LLLT group, n = 20) or placebo treatment (placebo group, n = 20). Patients in the LLLT group received LLLT twice a week for 3 weeks, for a total of 6 treatment sessions. The primary endpoint was the changes in the fluorescein corneal staining (FCS) score. The secondary endpoints were the changes in the ocular surface disease index (OSDI) score, lissamine green conjunctival staining (LGCS) scores, tear film break-up time (TBUT), Schirmer test, and the meibomian gland dysfunction (MGD) index. These were evaluated before treatment and 4 weeks after start of treatment. The mean difference of score change in primary endpoint revealed significant improvement in the LLLT group, compared to the placebo. Among secondary endpoints, LGCS, Schirmer's test, upper meibography scores showed significant improvements, while TBUT, lid debris, lid swelling, lid telangiectasia, meibomian gland secretion and expressibility scores had slight improvement without significant differences. No serious adverse events were observed. The use of LED-LLLT for the treatment of dry eye and MGD appears to be safe and beneficial.

## Introduction

Dry eye is common, affecting approximately 14–33% of the population which affects their work, their lifestyle and their comfort^1^. It causes visual disturbance and ocular discomfort. Tear hyperosmolarity, disruption of the ocular surface, and tear film instability induce dry eye syndrome^2^. Changed tear composition and decreased tear production result in damage to the ocular surface, inflammation, and apoptosis of epithelial cells^3^. The current treatments for dry eye are tear supplementation, tear stimulation, anti-inflammatory, and immunomodulatory medications^4^.

Low-level light therapy (LLLT) has been utilized in the treatment of various disorders. It induces biomodulation in the cellular metabolism as well as analgesic and anti-inflammatory effects^5^. Some in vivo and clinical studies have been made regarding the effect of LLLT on ocular surface or dry eye disease, and have proved the positive effect of LLLT for ophthalmic application^6–9^. However, information concerning the efficacy of LLLT in managing dry eye disease has been sparse and the parameters studied have been varied, mostly utilizing intense pulsed light along with LLLT. The present study was designed to investigate the efficacy and safety of LLLT using light-emitting diodes (LED-LLLT) in the treatment of patients with dry eye.

## Methods

### Ethics statement

This randomized clinical trial was conducted in accordance with the ethical principles set forth in the Declaration of Helsinki from April 2018 to April 2019. The study protocol and informed consent were reviewed and approved by the institutional review board (IRB) of Dankook university hospital before initiation (IRB#DKUH201802007). The study enrolled subjects at Dankook university hospital and subjects for this study were recruited in accordance with IRB standard for recruitment practices. Written informed consent was obtained from each patient before the start of the study. The name of the trial registry is CRIS (http://cris.nih.go.kr) and the registration number of the trial is KCT0004608 (date of registration: 07/01/2020). This study is reported per the Consolidated Standards of Reporting Trials (CONSORT) guidelines (S1 File).

### Study design and participants

A computer-generated list of random numbers was used to randomly assign patients with a 1:1 allocation ratio to receive LED-LLLT or placebo treatment. The randomization sequence was generated using permuted blocked randomization with block sizes of 2 and 4. Upon enrollment, each subject’s randomized assignment was displayed in the electronic data entry system and both the subject and the investigator were blinded after assignment to intervention. This trial was designed as a descriptive study with a planned enrollment of 20 subjects per group, including 20% drop-out rate. Sample size was determined with the general acceptance of 0.05 probability of Type 1 error and 80% power with an effect size of 0.8. It was assumed that the score difference of primary endpoint to be 1.5 and 0.5 in the LLLT and placebo group respectively, with standard deviation of 1^10^.

Eligible patients were 19 years of age or older who had been diagnosed as having dry eye syndrome and had dry eye-related symptoms that were present at the screening examination. Other inclusion criteria were: (1) a fluorescein corneal staining (FCS) score that revealed conjuctivo-corneal inflammation on the Oxford grading, (2) a Schirmer test value at 5 min of 10 mm or less, and (3) a tear film break up time (TBUT) value of 10 s or less.

Exclusion criteria included anterior ocular disease, patients who had a silicone lacrimal punctal occlusion within 3 months before the screening examination, patients who underwent refractive surgery like LASIK within 12 months or intraocular surgery within 3 months before the screening period, patients who used steroids, immunosuppressants, antihistamines or any other treatment affecting the dynamics of tear fluid, and history of a glaucoma, uveitis, blepharitis, Steven-Johnson syndrome or any other disease affecting the dynamics of tear fluid. Patients treated with additional dry eye therapies were excluded, as were pregnant or lactating females. Every patient received sodium hyaluronate ophthalmic suspension, 1 drop in each eye for 4–6 times daily during the treatment period.

### Assessment of outcome measures

The primary end point was the changes in the FCS score at week 4 (last observation). Secondary end points were the changes in the ocular surface disease index (OSDI) score^11^, the lissamine green conjunctival staining (LGCS) score, the Schirmer test value, expressible meibomian glands and quality, and TBUT. All of these parameters were evaluated at baseline, week 4 or at treatment discontinuation.

For the FCS scores, 5 μl of 2% fluorescein solution was instilled and FCS was examined using a slit-lamp microscope with a cobalt blue filter. TBUT was checked by the elapsed time from a normal blink to the first appearance of a dry spot and was measured 3 times. For LGCS scores, 20 μl of 1% lissamine green solution was applied as drops and conjunctival staining was evaluated by slit-lamp microscopy. Results were evaluated using the Oxford grading scheme from 0 to 6 with increased significance of ocular surface staining^12^. The Schirmer test was performed without anesthesia and the tear volume was measured for 5 min.

The meibum expressibility was scored as the value of meibum which could be induced. The quality of the meibum was graded as 1, clear; 2, cloudy; 3, thick like toothpaste; and 4, no meibum. Lid debris, lid swelling, and lid telangiectasia were scored from 0 (absent) through 4 (very severe) according to the severity of these abnormalities present in each eye. Using the noncontact meibography system (Meiboviewer, Visual optics, Choonchun, Korea) the meibomian glands were observed. The dropout of the meibomian glands was graded as 0 (no dropout), 1 (dropout of meibomian glands < 1/3), 2 (1/3 < dropout of meibomian glands < 2/3), and 3 (dropout of meibomian glands > 2/3). Meibography scores were evaluated from 0 through 3 for the upper and lower eyelids of each eye. The safety variable was the occurrence of adverse events by slit-lamp microscopy and funduscopy.

### LLLT protocol

LLLT was conducted to the patients at Dankook University Hospital upon the scheduled visits of each patient. Light irradiation was performed with a LED-based array matrix module with 5 planar panels. For the first 1 min of irradiation a wavelength of 590 nm in continuous wave and an irradiance of ≈ 50 µW/cm^2^ scanned panel by panel for 1 s per panel, giving a fluence of ≈ 50 µJ/cm^2^. At the end of that minute, 830 nm was delivered from all 5 panels at an irradiance of 100 mW/cm^2^ for 10 min in continuous wave mode. The system is LED-based, and the irradiance was below the ANSI values for maximum permissible exposure (MPE). The energy density over the entire near field area (encompassing the entire face and orbital area) was therefore ≈ 60 J/cm^2^ per session (HEALITE II, Lutronic, Goyang, Korea), with a distance between the treatment head and target tissue of approximately 17 cm.

Reports have suggested that the above parameters with the 830 nm LED-based approach have been safe and effective in general for wound healing and as an adjunctive approach to other aesthetic procedures^13,14^. Specifically, the same device as used in the present study has been proven safe and effective for treating wounds on the face around the orbit, which incidentally included irradiation of the closed eyes^15^, and for effective and safe treatment of acute herpes zoster ophthalmicus which would obviously include the orbital area^16^. Taken in conjunction with existing literature on the treatment of dry eye, the parameters in the current study were selected.

The system targeted both the closed lids (upper and lower) and periorbital area. Patients in the LLLT group received LED-LLLT twice a week for 3 weeks, for a total of 6 treatment sessions. Light-occlusive metal contact lenses inserted following lubricating drops protected the patients’ eyes during the procedure. Placebo treatment was performed with a system which looked and sounded identical in every way to the active system, following the same protocol used for irradiated patients, but with no active LED energy emission.

### Statistical analysis

Statistical methods and data analysis were performed using SPSS version 19.0, and the effect size calculation was done on a web calculator^17^. The mean differences in the primary and secondary endpoints between the LLLT and placebo groups at week 4 were analyzed. Additionally, the mean differences in score changes from baseline to week 4 (posttest score–pretest score) between the groups were compared.

The distribution of the data was checked for normality using the Shapiro–Wilk test. Depending on the data distribution, independent sample t-test and Wilcoxon rank sum test were used for parametric and non-parametric data, respectively. The data are presented as the mean and standard deviation, with respective 95% confidence interval and effect sizes. Categorical variables are presented as proportions (%) and were compared using Fisher’s exact test or the chi-squared test. A p-value of < 0.05 was considered to be statistically significant. Analysis of outcome was based on intention-to-treat (ITT) analysis including all patients, and per-protocol (PP) analysis with only those who received treatment excluding the patients with discontinued interventions.

## Results

### Participant characteristics

A total of 40 patients were enrolled in the study between October 17, 2018 and February 8, 2019 and randomly assigned to receive LED-LLLT (LLLT group, n = 20) or placebo treatment (placebo group, n = 20). In placebo group, 2 patients refused to continue due to ocular discomfort, and 20 patients were analyzed with ITT analysis and 18 patients without the discontinued patients were analyzed with PP analysis. Demographics and other baseline characteristics are shown in Table 1.

**Table 1:** Baseline demographic and clinical characteristics of each group.

	LLLT group (n = 20)	Placebo group (n = 20)
**Characteristic**		
Age, mean (SD)	46.0 (13.5)	42.3 (14.1)
Male (sex), no. (%)	3 (15%)	5 (25%)
**Primary endpoint, mean (SD)**		
Fluorescein corneal staining (FCS)	2.55 (1.20)	1.95 (1.47)
**Secondary endpoints, mean (SD)**		
Ocular surface disease index (OSDI)	36.64 (19.51)	37.37 (16.91)
Tear break-up time (TBUT)	5.33 (1.01)	5.70 (1.36)
Lissamine green conjunctival staining (LGCS)	1.60 (1.20)	0.85 (1.01)
Schirmer’s test	6.85 (4.73)	8.38 (4.76)
Lid debris	1.30 (1.31)	1.5 (1.07)
Lid swelling	1.40 (1.43)	1.70 (1.45)
Lid telangiectasia	2.40 (1.36)	2.60 (1.28)
Meibomian gland quality	2.00 (1.22)	2.00 (0.63)
Meibomian gland expressibility	11.30 (2.74)	10.75 (2.17)
Upper eyelid meibography score	2.00 (1.18)	1.90 (1.41)
Lower eyelid meibography score	2.30 (0.90)	2.40 (1.16)

### Efficacy evaluation

Mean differences in the primary and secondary endpoints at week 4 were compared between the LLLT and placebo groups by ITT and PP analysis.

ITT analysis on the primary and secondary endpoints in LLLT and placebo groups showed no significant differences among the variables (Table 2). The primary endpoint, the mean FCS score (SD) at week 4 in the LLLT and placebo groups were 1.45 (1.39) and 1.55 (1.24) respectively, being slightly lower in LLLT group without significant difference (p = 0.717). Among the secondary endpoints, the mean (SD) of lid debris (0.65 (1.04), 1.10 (0.99); p = 0.164), lid swelling (0.70 (0.98), 1.20 (1.33); p = 0.250), lid telangiectasia (1.55 (0.83), 1.90 (1.34); p = 0.426), meibomian gland quality (1.75 (0.79), 1.80 (0.87); p = 0.985), upper eyelid meibography (1.30 (1.26), 1.45 (1.36); p = 0.764) and lower eyelid meibography (1.75 (1.25), 1.90 (1.14); p = 0.823) scores were all higher in placebo groups, having small to medium effect sizes (0.241–0.576). The mean (SD) of TBUT (6.84 (1.88), 5.97 (2.71); p = 0.256), Schirmer’s test (9.03 (4.41), 7.75 (5.13); p = 0.412) and meibomian gland expressibility (12.85 (1.75), 10.85 (4.05); p = 0.137) were higher in LLLT group, having small to medium effect sizes (0.094–0.433) respectively.

**Table 2:** Intention-to-treat analysis for the primary and secondary endpoints after LLLT interventions.

	LLLT group (n = 20)	95% CI (LLLT)	Placebo group (n = 20)	95% CI (placebo)	Effect size (*d*_cohen_)	p-value
**Primary endpoint**						
Fluorescein corneal staining (FCS)^a^	1.45 (1.39)	0.80–2.10	1.55 (1.24)	0.95–2.15	0.211	0.717
**Secondary endpoints**						
Ocular surface disease index (OSDI)^b^	25.20 (19.29)	16.17–34.23	24.05 (17.80)	15.21–31.97	0.035	0.787
Tear break-up time (TBUT)^b^	6.84 (1.88)	5.96–7.73	5.97 (2.71)	4.67–7.28	− 0.106	0.256
Lissamine green conjunctival staining (LGCS)^a^	0.85 (0.99)	0.39–1.31	0.60 (0.80)	0.22–0.98	− 0.202	0.495
Schirmer’s test^b^	9.03 (4.41)	6.96–11.09	7.75 (5.13)	5.29–10.21	− 0.094	0.412
Lid debris^a^	0.65 (1.04)	0.16–1.14	1.10 (0.99)	0.62–1.58	0.576	0.164
Lid swelling^a^	0.70 (0.98)	0.24–1.16	1.20 (1.33)	0.56–1.84	0.545	0.250
Lid telangiectasia^a^	1.55 (0.83)	1.16–1.94	1.90 (1.34)	1.26–2.54	0.537	0.426
Meibomian gland quality^a^	1.75 (0.79)	1.38–2.12	1.80 (0.87)	1.38–2.22	0.346	0.985
Meibomian gland expressibility^a^	12.85 (1.75)	12.03–13.67	10.85 (4.05)	8.90–12.80	− 0.433	0.137
Upper eyelid meibography^a^	1.30 (1.26)	0.71–1.89	1.45 (1.36)	0.80–2.10	0.241	0.764
Lower eyelid meibography^a^	1.75 (1.25)	1.16–2.34	1.90 (1.14)	1.35–2.45	0.324	0.823

^a^Wilcoxon rank sum test results.

^b^Independent sample t-test.

PP analysis on the primary and secondary endpoints in the LLLT and placebo groups showed no significant differences among the variables (Table 3). The primary endpoint, the mean FCS score (SD) at week 4 in the LLLT and placebo groups were 1.45 (1.39) and 1.72 (1.23) respectively, being lower in LLLT group without significant difference (p = 0.417). Among the secondary endpoints, the mean (SD) of OSDI (25.20 (19.29), 26.22 (16.91); p = 0.815), lid debris (0.65 (1.04), 1.22 (1.00); p = 0.090) , lid swelling (0.70 (0.98), 1.33 (1.37); p = 0.143), lid telangiectasia (1.55 (0.83), 2.11 (1.28); p = 0.125), meibomian gland quality (1.75 (0.79), 2.00 (0.68); p = 0.420), upper eyelid meibography (1.30 (1.26), 1.61 (1.38); p = 0.488) and lower eyelid meibography (1.75 (1.25), 2.11 (1.02); p = 0.462) scores were all higher in placebo groups, having small to medium effect sizes (0.235–0.599). The mean (SD) of TBUT (6.84 (1.88), 6.64 (2.00); p = 0.749), Schirmer's test (9.03 (4.41), 8.61 (4.80); p = 0.714) and meibomian gland expressibility (12.85 (1.75), 12.06 (1.98); p = 0.312) were higher in LLLT group, having small to medium effect sizes (0.091–0.423) respectively.

**Table 3:** Per-protocol analysis for the primary and secondary endpoints after LLLT interventions.

	LLLT group (n = 20)	95% CI (LLLT)	Placebo group (n = 18)	95% CI (placebo)	Effect size (*d*_cohen_)	p-value
**Primary endpoint**						
Fluorescein corneal staining (FCS)^a^	1.45 (1.39)	0.80–2.10	1.72 (1.23)	1.11–2.33	0.205	0.417
**Secondary endpoints**						
Ocular surface disease index (OSDI)^a^	25.20 (19.29)	16.17–34.23	26.22 (16.91)	17.81–34.63	0.056	0.815
Tear break-up time (TBUT)^b^	6.84 (1.88)	5.96–7.73	6.64 (2.00)	5.65–7.73	− 0.103	0.749
Lissamine green conjunctival staining (LGCS)^a^	0.85 (0.99)	0.39–1.31	0.67 (0.84)	0.25–1.08	− 0.195	0.495
Schirmer’s test^a^	9.03 (4.41)	6.96–11.09	8.61 (4.80)	6.22–11.00	− 0.091	0.714
Lid debris^a^	0.65 (1.04)	0.16–1.14	1.22 (1.00)	0.72–1.72	0.558	0.090
Lid swelling^a^	0.70 (0.98)	0.24–1.16	1.33 (1.37)	0.65–2.02	0.545	0.143
Lid telangiectasia^a^	1.55 (0.83)	1.16–1.94	2.11 (1.28)	1.48–2.75	0.534	0.125
Meibomian gland quality^a^	1.75 (0.79)	1.38–2.12	2.00 (0.69)	1.66–2.34	0.336	0.420
Meibomian gland expressibility^a^	12.85 (1.75)	12.03–13.67	12.06 (1.98)	11.07–13.04	− 0.424	0.312
Upper eyelid meibography^a^	1.30 (1.26)	0.71–1.89	1.61 (1.38)	0.93–2.30	0.235	0.488
Lower eyelid meibography^a^	1.75 (1.25)	1.16–2.34	2.11 (1.02)	1.60–2.62	0.314	0.462

^a^Wilcoxon rank sum test results.

^b^Independent sample t-test.

Considering the differences in the primary and secondary endpoints between the LLLT and placebo groups at baseline, the mean differences of score changes at week 4 (post–pre test scores) were as well analyzed by ITT and PP.

ITT analysis on the score changes of primary and secondary endpoints in LLLT and placebo groups showed significant differences among the variables (Table 4). The primary endpoint, the mean FCS score change (SD) at week 4 in the LLLT and placebo groups showed significant differences, being − 1.10 (1.07) and − 0.40 (1.16) respectively (p = 0.028), having a medium effect size (0.627). Among the secondary endpoints, the mean LGCS score change (− 0.75 (1.12), − 0.25 (0.62); p = 0.029), Schirmer's test score change (2.18 (3.17), − 0.63 (3.25); p = 0.005), and upper eyelid meibography score change (− 0.70 (0.80), − 0.45 (1.20); p = 0.023) in LLLT and placebo groups also showed significant differences, with small to large effect sizes (0.247–0.886). The LLLT group showed greater decrease in TBUT, lid debris, lid swelling, lid telangiectasia, meibomian gland quality, meibomian gland expressibility, and lower eyelid meibography score changes did not show significant differences.

**Table 4:** Intention-to-treat analysis for the score changes of primary and secondary endpoints after LLLT interventions.

	LLLT group^a^ (n = 20)	95% CI (LLLT)	Placebo group^a^ (n = 20)	95% CI (placebo)	Effect size (*d*_cohen_)	p-value
**Primary endpoint**						
Fluorescein corneal staining (FCS)^b^	− 1.10 (1.07)	− 1.60 to 0.60	− 0.40 (1.16)	− 0.96 to 0.16	0.627	0.028
**Secondary endpoints**						
Ocular surface disease index (OSDI)^b^	− 11.45 (12.93)	− 17.50 to − 5.40	− 13.55 (20.24)	− 23.27 to − 3.83	− 0.125	0.503
Tear break-up time (TBUT)^c^	1.51 (1.53)	0.80 to 2.22	0.27 (2.93)	− 0.62 to 1.63	− 0.534	0.092
Lissamine green conjunctival staining (LGCS)^b^	− 0.75 (1.12)	− 1.27 to − 0.23	− 0.25 (0.62)	− 0.55 to 0.05	0.564	0.029
Schirmer’s test^b^	2.18 (3.17)	0.69 to 3.66	− 0.63 (3.25)	− 2.60 to 0.88	− 0.886	0.005
Lid debris^b^	− 0.65 (1.23)	− 1.22 to − 0.08	− 0.40 (1.02)	− 0.89 to 0.09	0.226	0.259
Lid swelling^b^	− 0.70 (0.98)	− 1.16 to − 0.24	− 0.50 (1.07)	− 1.01 to 0.01	0.198	0.240
Lid telangiectasia^b^	− 0.85 (1.18)	− 1.40 to − 0.30	− 0.70 (1.14)	− 1.25 to − 0.15	0.131	0.336
Meibomian gland quality^b^	− 0.25 (0.91)	− 0.68 to 0.18	− 0.20 (0.60)	− 1.95 to 0.35	0.066	0.368
Meibomian gland expressibility^b^	1.55 (1.76)	0.73 to 2.37	0.10 (3.06)	− 0.12 to 1.52	− 0.584	0.050
Upper eyelid meibography^b^	− 0.70 (0.80)	− 1.08 to − 0.33	− 0.45 (1.20)	− 1.03 to 0.13	0.247	0.023
Lower eyelid meibography^b^	− 1.00 (1.12)	− 0.99 to − 0.11	− 0.50 (1.36)	− 1.15 to 0.15	0.043	0.131

^a^Post-Pre test scores.

^b^Wilcoxon rank sum test results.

^c^Independent sample t-test.

PP analysis on the score changes of primary and secondary endpoints in LLLT and placebo groups showed significant differences among the variables (Table 5). The primary endpoint, the mean FCS score change (SD) at week 4 in the LLLT and placebo groups showed significant differences, being − 1.10 (1.07) and − 0.22 (1.11) respectively (p = 0.017), having a large effect size (0.808). Among the secondary endpoints, the mean LGCS score change (− 0.75 (1.12), − 0.28 (0.70); p = 0.043), Schirmer's test score change (2.18 (3.17), − 0.11 (3.07); p = 0.031), upper eyelid meibography score change (− 0.70 (0.80), − 0.05 (0.24); p = 0.003), and lower eyelid meibography score change (− 1.00 (1.12), − 0.56 (1.49); p = 0.046) in LLLT and placebo groups also showed significant differences, with medium to large effect sizes (0.336–1.076). Even with greater decrease in LLLT group, OSDI, TBUT lid debris, lid swelling, lid telangiectasia, meibomian gland quality and meibomian gland expressibility score changes did not show significant differences.

**Table 5:** Per-protocol analysis for the score changes of primary and secondary endpoints after LLLT interventions.

	LLLT group^a^ (n = 20)	95% CI (LLLT)	Placebo group^a^ (n = 18)	95% CI (placebo)	Effect size (*d*_cohen_)	p-value
**Primary endpoint**						
Fluorescein corneal staining (FCS)^b^	− 1.10 (1.07)	− 1.60 to 0.60	− 0.22 (1.11)	− 0.78 to 0.33	0.808	0.017
**Secondary endpoints**						
Ocular surface disease index (OSDI)^c^	− 11.45 (12.93)	− 17.50 to − 5.40	− 8.22 (13.34)	− 14.86 to − 1.59	0.246	0.454
Tear break-up time (TBUT)^c^	1.51 (1.53)	0.80 to 2.22	1.14 (1.41)	0.44 to 1.85	− 0.251	0.448
Lissamine green conjunctival staining (LGCS)^b^	− 0.75 (1.12)	− 1.27 to − 0.23	− 0.28 (0.70)	− 0.61 to − 0.− 06	0.497	0.043
Schirmer’s test^c^	2.18 (3.17)	0.69 to 3.66	− 0.11 (3.07)	− 1.64 to 1.42	− 0.733	0.031
Lid debris^b^	− 0.65 (1.23)	− 1.22 to − 0.08	− 0.11 (0.47)	− 0.35 to 0.12	0.568	0.138
Lid swelling^b^	− 0.70 (0.98)	− 1.16 to − 0.24	− 0.22 (0.65)	− 0.45 to − 0.10	0.571	0.088
Lid telangiectasia^b^	− 0.85 (1.18)	− 1.40 to − 0.30	− 0.44 (0.86)	− 0.87 to − 0.02	0.394	0.250
Meibomian gland quality^b^	− 0.25 (0.91)	− 0.68 to 0.18	−	−	0.378	0.174
Meibomian gland expressibility^b^	1.55 (1.76)	0.73 to 2.37	1.00 (0.157)	0.22 to 1.78	− 0.429	0.239
Upper eyelid meibography^b^	− 0.70 (0.80)	− 1.08 to − 0.33	− 0.05 (0.24)	− 0.17 to 0.06	1.076	0.003
Lower eyelid meibography^b^	− 1.00 (1.12)	− 0.99 to − 0.11	− 0.56 (1.49)	− 0.17 to 0.06	0.336	0.046

^a^Post-Pre test scores.

^b^Wilcoxon rank sum test results.

^c^Independent sample t-test.

### Safety evaluation

No deaths and no serious adverse events were observed in this study.

## Discussion

This study evaluated the effect of light-emitting diode-based LLLT (LED-LLLT) on a group of patients with dry eye disease and found positive results on improving symptoms. Dry eye patients often complain of blurriness and glare even though the result of visual acuity is normal^18^. The tear film breakup, tear hyperosmolarity, ocular surface inflammation, which causes an irregular tear surface, may be the cause of visual deterioration^19^. Meibomian gland dysfunction (MGD) has proved to be a risk factor for dry eye^20^. The gland dropouts with age, obstruction at the opening of duct, chronic damage, and poor quality meibum are associated with MGD^21^.

LLLT in general, or photobiomodulation, is a treatment approach currently used for dermatological and other medical purposes. The biological action of LLLT is thought to take place through the intracellular absorption of energy by the cellular membrane, intracellular organelles and molecules depending on the wavelength. Athermal and atraumatic cellular photoactivation occurs with light emitting diodes of specific wavelengths. This photoactivation is shown to repair damaged or compromised cells and improve cellular function in normal cells^13^. The chief medical indication for LLLT are in accelerating and enhancing tissue repair and promoting regeneration of a variety of tissues and nerves, reducing pain and inflammation, and preventing tissue damage^22^. LLLT in the present study comprised exposing the eyelid tissue to low-levels of yellow and near infrared light. The selection of laser parameters depends on the application target. The optical features of tissues differ markedly and are characterized by scattering and absorption coefficients, which depend on wavelength^23^.

Although LLLT has been historically performed using mostly laser-based light sources, the past decade has seen an increasing number of reports on LLLT delivered with light-emitting diode (LED) arrays (LED-LLLT). LED-LLLT offers advantages over laser diode (LD)-LLLT. Although LEDs are noncoherent, good quality LEDs are quasimonochromatic, with more than 98% of the photons at the rated wavelength, and the construction of the LED chips ensures good directionality with photons all travelling in approximately the same direction, albeit out of phase: this means that LEDs cannot be collimated or focused to a point. Secondly, because they are noncoherent and deliver a divergent beam of photon energy, LEDs are intrinsically safer than LDs because the pupil can gather only a minute fraction of the emitted noncoherent light, and as already mentioned LED energy cannot be focused to a point because LEDs are not a point source, which LDs are. Finally, LEDs can be mounted in planar arrays, thereby enabling irradiation of large areas of tissue in a hands-free manner, delivering a large area of homogeneous near-field irradiance and making precise positioning of the array over the target less necessary.

The skin chromophores (blood and melanin) possess high absorption bands at wavelengths shorter than 610 nm, therefore visible light does not penetrate deeply into the dermis. In the visible band from 610 to 700 nm, penetration increases by approximately 5 orders of magnitude, and at the wavelength of 830 nm, penetration is deepest before water becomes a major chromophore at wavelengths greater than 1150 nm. This results in the so-called optical window that covers the red and near infrared wavelengths and in which the effective tissue penetration of light is maximized^24^. Phototherapy utilizes light wavelengths between 390 and 1100 nm and may be pulsed or continuous wave. In standard condition, relatively low power densities (< 100 mW/cm^2^) are utilized^25^. To treat superficial tissue, wavelengths in the range of 390 nm to 600 nm are applied and longer wavelengths in the range of 600 nm to 1100 nm are applied to treat deeper tissues^26^. LLLT in the red to near infrared spectral range (630–1000 nm) and nonthermal power (less than 200 mW) is known to be effective for treatment of acne vulgaris^27^. A previous study evaluated the efficacy of a combination of 830 nm and 630 nm wavelengths in two sessions over 4 or 5 weeks to treat recalcitrant psoriasis employing LED irradiation and revealed no adverse side effects and a resolution of psoriasis^28^. A LED-based matrix module operating at the main wavelength of 830 nm, as utilized in our study, provided the effective penetration depth and was well tolerated on eye tissues^14^. The 590 nm component applied in the present study for the first minute of the total 11-min treatment time has as its targets the mother keratinocytes in the stratum basale in the epidermis, releasing adenosine triphosphate into the epidermal matrix and preconditioning the dermis. The subsequent 830 nm energy literally ‘washes out’ the clinical efficacy of the 590 nm energy at more than 3 orders of magnitude greater irradiance, and therefore targets all cells in the eyelids, including the meibomian glands. The combination of the effects of these wavelengths in the system used in the present study has proven highly effective in wound healing and skin rejuvenation, with particular efficacy accorded to the main wavelength of 830 nm^29^. With the combination of the inherent safety associated with noncoherent diffuse LED energy and the use of the metal contact eyeshields, no ocular safety issues were raised, and no serious adverse events occurred during treatment or the follow-up period.

In past few years, LLLT has been applied on dry eye patients to improve the symptoms and lower the severity of disease^7–9^. However, most of them were applied along with intense pulsed light (IPL) with positive effects, which makes it difficult to conclude the effect of LLLT itself. To our knowledge, this is the first randomized controlled trial to evaluate the sole effects of LED-LLLT on the treatment of dry eye disease^30^. Our study has shown a decrease in dry eye symptom, suggesting that LLLT can be used for lacrimal and meibomian gland alterations. LLLT is widely used by dermatologists, plastic surgeons and other specialties due to its analgesic, anti-inflammatory, and biostimulatory effects, and recent reports have shown the efficacy of a combination of LLLT with intense pulsed light (IPL) therapy for meibomian gland dysfunction, and LLLT on its own in the treatment of chalazia^7,14,31^. Evidence has therefore started to accrue for the safety and efficacy of LLLT on ophthalmic tissues, and we believe that our study has added to that evidence. Due to the fact that FCS reflects corneal damage and LGCS reflects conjunctival epithelium integrity, these improvements at week 4 in the present study revealed that LED-LLLT improved the ocular surface conditions. Schirmer’s test results in the LLLT group showed a significant improvement at week 4 compared to the placebo group. This could be the result of the well-proven anti-inflammatory properties of LLLT indirectly leading to tear secretion. Also, improvement in upper eyelid meibography was observed in the LLLT group. There were more meibomian gland dropouts in the lower eyelid than upper eyelid before the intervention, so it may be the reason why lower meibography score did not show inter-group difference. Since the area of upper eyelid is wider than lower eyelid, it seems that the photobiomodulatory effect of LLLT is efficient. LLLT delivered with a light-emitting diode array may be effective for dry eye syndrome by stimulating the function of both the lacrimal glands and meibomian glands^6^.

Our study as well revealed that there have been slight improvements in TBUT, lid debris, lid swelling, lid telangiectasia, meibomian gland quality, meibomian gland expressibility scores in the LLLT group compared to the placebo group, having greater mean difference of score changes after interventions. The tendency of these improvements can be correlated with the beneficial photobiomodulatory effects of particularly the 830 nm components. However, such slight difference between the groups could have been the effect of the concomitant use of sodium hyaluronate eye drop. Artificial tears are frequently used with anti-inflammatory or immunomodulatory ophthalmic solutions in clinical practice^32^.

The results of the current study revealed the positive effect of LED-LLLT on patients with dry eye, however there are still a few limitations in this study. First, the effect of LED-LLLT on dry eye with statistically significant differences were only observed among the mean differences of score changes in the primary and secondary endpoints. Upon random allocation of participants into two equal arms, some of the endpoint scores at baseline in the LLLT and placebo groups already showed small to medium effect sizes (0–0.676). Second, the current study confirmed the improvement of symptoms during the trial period, and follow-up results were not considered in analysis. Third, this study showed no significant differences between the improvement of symptoms and age or sex. Considering that the severity of MGD is related with the age and sex of patients, subsequent studies on a greater number of patients may be required.

In conclusion, this is the first study to investigate the efficacy and safety of LLLT with LEDs in patients with dry eye syndrome. Our study demonstrated that LED-LLLT at a dose of ≈ 60 J/cm^2^ per treatment session effected improvements in the signs and symptoms of dry eye. Therefore, our study revealed that LLLT applied as a treatment for dry eye can stimulate lacrimal gland and meibomian gland function. Such efficacy, in addition to the well-tolerated profile of LLLT, makes it a potentially useful treatment option for dry eye in clinical practice.

## Supplementary Information

Supplementary Information.

## Data Availability

All data generated or analyzed during this study are included in this article.
